# REDUCED LEVELS OF NAD IN SKELETAL MUSCLE IN PEOPLE AGING WITH HIV INFECTION ON TREATMENT

**DOI:** 10.1093/geroni/igac059.2453

**Published:** 2022-12-20

**Authors:** Monty Montano, Karol Pencina, Michael Schultz, Thanh Tran, Zhuoying Li, Catherine Ghattas, Jackson Lau, David Sinclair

**Affiliations:** Harvard Medical School, Boston, Massachusetts, United States; Harvard Medical School, Boston, Massachusetts, United States; Harvard Medical School, Boston, Massachusetts, United States; Boston University, Boston, Massachusetts, United States; Harvard Medical School, Boston, Massachusetts, United States; Brigham and Women's Hospitial, Boston, Massachusetts, United States; Brigham and Women's Hospital, Boston, Massachusetts, United States; Harvard Medical School, Boston, Massachusetts, United States

## Abstract

People living with HIV (PLWH) are disproportionately burdened with multimorbidity and decline in physiologic function compared to their uninfected counterparts, but biological mechanisms that differentially contribute to the decline in muscle function in PLWH compared to uninfected people remains understudied. The study site was Brigham and Women’s Hospital, Harvard Medical School, Boston MA. We evaluated skeletal muscle tissue for levels of total NAD, NAD+ and NADH in middle-aged asymptomatic PLWH, coinfected with hepatitis C virus (HCV) and/or cytomegalovirus (CMV) and compared them to uninfected control participants. Of the 54 persons with muscle biopsy data, the mean age 57 years with 33% women. Total NAD levels declined in skeletal muscle in association with HIV infection, and was exacerbated by HCV and CMV coinfection, with lowest levels of total NAD, NAD+ and NADH among persons that were coinfected with all three viruses (P=0.015, P=0.014, P=0.076; respectively). Levels of total NAD, NAD+ and NADH in skeletal muscle were inversely associated with inflammation (P=0.014, P=0.013, P=0.055; respectively). Coinfections were also associated with measures of inflammation (CD4/CD8 ratio: P< 0.001 and sCD163: P< 0.001), immune activation (CD38 and HLA-DR expression on CD8 T cells: P< 0.001). Additionally, coinfection was associated with increased physiologic frailty, based on the VACS Index 1.0 assessment (P=0.001). Further research is warranted to determine the clinical relevance of preclinical deficits in NAD metabolites in skeletal muscle in association with viral coinfection and inflammation, as well as the observed association between viral coinfection and physiologic frailty.

